# Cognitive deficits linked to intrinsic timescales and gray matter volume abnormalities in children with Duchenne muscular dystrophy

**DOI:** 10.1186/s11689-026-09689-x

**Published:** 2026-03-29

**Authors:** Xiaoyu Niu, Qin Hu, Xinyuan Zhang, Suming Zhang, Ke Xu, Rong Xu, Yu Song, Hang Fu, Ziqi Zhou, Ying Ren, Caihan Li, Ting Xu, Shaoqiang Han, Yong Zhang, Huayan Xu, Xiaotang Cai, Bochao Cheng, Yingkun Guo

**Affiliations:** 1 Department of Radiology, Medical Imaging Center, West China Second University Hospital, Sichuan University, Chengdu, China; 2 Department of Rehabilitation Medicine, West China Second University Hospital, Sichuan University, Chengdu, Sichuan China; 3 Department of Radiology, Huaxi MR Research Center (HMRRC), West China Hospital of Sichuan University, Chengdu, China; 4 Department of Magnetic Resonance Imaging, First Affiliated Hospital of Zhengzhou University, Zhengzhou, China

**Keywords:** Duchenne muscular dystrophy, Intrinsic neural timescale, Gray matter volume, Cognitive deficits, Network dysfunction

## Abstract

**Background:**

Duchenne muscular dystrophy (DMD) is associated with cognitive deficits and neural abnormalities, yet how temporal properties of regional brain activity align with structural changes across development remains unclear. We integrated intrinsic neural timescale (INT) with voxel-based morphometry (VBM) to explore whether intrinsic timescales disruptions co-localize with gray matter volume (GMV) alterations and relate to cognition in children with DMD.

**Methods:**

Thirty-six children with DMD and 30 age-matched healthy controls underwent T1-weighted MRI and resting-state fMRI. INT and VBM analyses were conducted to assess intrinsic timescales and GMV, respectively. Group differences were tested in statistical parametric mapping toolkit using two-sample *t*-tests with Gaussian random-field correction (voxel-wise *P* < 0.001; cluster-wise *P* < 0.05). Global volumetrics were analyzed with ordinary least squares models including group × age interactions. Spearman rank correlations were subsequently computed to assess associations between cognitive scores and the identified neural abnormalities.

**Results:**

DMD showed cognitive impairment and distinct neurodevelopmental features, including: co-localized shorter INT and lower GMV within limbic–sensorimotor networks; widespread GMV reductions across the visual, default-mode, and dorsal attention networks; and divergent age-related trends in global volumes. Moreover, GMV in multiple abnormal regions correlated positively with working memory and perceptual reasoning scores.

**Conclusions:**

These findings suggest that dystrophin deficiency induces co-located functional-structural deficits and aberrant neurodevelopmental trends, offering insights into neurodevelopmental abnormalities in children with DMD. The integration of INT and GMV provides a novel framework for decoding hierarchical network dysfunction and morphological plasticity changes in DMD, identifying potential targets for cognitive intervention.

## Introduction

Duchenne muscular dystrophy (DMD), an X-linked recessive disorder caused by mutations in the dystrophin gene, affects approximately 1 in 3,500-5,000 male births and results in a lack of functional dystrophin protein [1, 2]. Dystrophin isoforms are expressed in muscle, along with cortical neurons and cerebellar Purkinje cells [3], suggesting involvement in neurocognitive functions [4, 5]. Individuals with DMD typically exhibit a full-scale intelligence quotient (FSIQ) approximately one standard deviation below the population mean [6]. Cognitive deficits in attention, executive function and memory are frequently observed [4]. These findings indicate widespread neural dysfunction; however, the neurobiological mechanisms linking dystrophin deficiency to cognitive impairment remain unclear, impeding development of targeted interventions.

In pediatric DMD cohorts, functional magnetic resonance imaging (fMRI) studies have revealed distinct neural alterations. These include aberrant hyperactivation within the default mode network (DMN) and executive control network (ECN), as well as suppressed activity in somatosensory and cerebellar-visual circuits, which have been correlated with cognitive profiles [7, 8]. Furthermore, region-specific gray matter volume (GMV) reductions, particularly in the insula, occipital lobes, and cerebellum, have been identified [9]. However, most existing work has focused on resting-state functional connectivity or morphological changes, and little is known about how the temporal properties of regional brain activity are altered in DMD during development.

The intrinsic neural timescale (INT) is a recently proposed metric that quantifies how long local neural activity remains self-predictive—i.e., in resting-state fMRI, the duration over which a region’s blood oxygenation level dependent signal stays correlated with itself. Shorter INT reflects faster, more transient dynamics and weaker temporal integration, whereas longer INT reflects more sustained dynamics and stronger temporal integration [10–12]. Specialization and hierarchical organization are fundamental aspects of primate cortical architecture [13]. At the whole-cortex level, INTs follow a hierarchical gradient—primary sensory cortices exhibit short INTs and high temporal precision, whereas association cortices (frontoparietal, temporal, and default-mode networks) exhibit long INTs that enable information integration across longer temporal windows [13–15]. This hierarchical organization offers a priori context for interpreting INT abnormalities in neurodevelopmental disorders.

Brain dystrophin deficiency in DMD—and downstream alterations in synaptic signaling—have been reported, particularly within association networks supporting higher-order cognition [16, 17]. These pathophysiological processes are expected to impact the temporal stability of regional activity. INT provides a time-domain characterization of this stability and complements conventional resting-state metrics that focus on spatial coupling. Accordingly, INT may serve as a sensitive time-domain biomarker for detecting hierarchy-related network vulnerabilities in DMD during childhood and adolescence. Theory and evidence indicate that the intrinsic timescale gradient relates to structural properties such as synaptic density [18, 19]. Given that dystrophin deficiency in DMD compromises neuronal cytoskeleton and synaptic plasticity [2, 20], INT and GMV are likely to be impacted simultaneously. However, it remains unclear whether the temporal properties of regional brain activity are altered in children with DMD, whether such alterations co-localize with GMV abnormalities, and how they relate to cognitive performance.

To address these gaps, the current study evaluated INT gradients and GMV in a pediatric DMD cohort and examining how abnormalities relate to cognitive performance. Based on prior work [8, 9, 11], we hypothesized that, relative to healthy controls (HCs), children with DMD would show region-specific GMV reductions (e.g., cerebellum, limbic system, and default mode network) and shortened INT in cognition-related regions, with partial spatial overlap between INT and GMV abnormalities.

## Materials and methods

### Participants

This study enrolled 42 male children with genetically confirmed DMD from the Neurorehabilitation Department of West China Second University Hospital. A group of 30 age- and sex-matched HCs was also enrolled. Both groups met the following inclusion criteria: (1) male sex and age < 18 years; (2) absence of neuropsychiatric disorders, neurosensory deficits, physical illnesses, or substance dependence. The exclusion criteria were: (1) recent use of antipsychotic or sedative medications and (2) contraindications for MRI.

This study adhered to the principles of the Declaration of Helsinki and received ethical approval from the Medical Research Ethics Committee of West China Second University Hospital, Sichuan University (approval number 2024392). Written informed consent was obtained from all participants or their legal guardians.

### Data acquisition

#### Neurodevelopmental assessment

Cognitive function was assessed in all participants using the Wechsler Intelligence Scale for Children-Fourth Edition (WISC-IV), which evaluated FSIQ, as well as the verbal comprehension index (VCI), perceptual reasoning index (PRI), working memory index (WMI), and processing speed index (PSI) [8, 21].

#### MRI scanning

MRI data were acquired using a 3T Siemens MAGNETOM Skyra scanner (Siemens Healthcare, Erlangen, Germany) equipped with a 32-channel head coil. Participants remained supine, awake, and with eyes closed during scanning, while foam padding and earplugs minimized motion and noise. Structural images were obtained using a three-dimensional magnetization-prepared rapid gradient echo (3D-MPRAGE) sequence with the following parameters: repetition time/echo time = 2000/2.19 ms, field of view = 256 × 224 mm^2^, sagittal slices = 176, slice thickness = 1.0 mm, skip = 0 mm, and flip angle = 9°. Functional images were acquired using a gradient echo-planar imaging sequence with the following parameters: repetition time/echo time = 2000/30 ms, flip angle = 90°, field of view = 240 × 240 mm^2^, voxel size = 3.75 × 3.75 × 5 mm^3^, slices = 28, slice thickness = 5.0 mm, and a total of 200 volumes.

### Data analysis

#### Functional data preprocessing and intrinsic timescale map

Functional images were preprocessed using the Data Processing Assistant for Resting-State fMRI (DPARSF), built upon the framework of Statistical Parametric Mapping 12 (SPM 12) and MATLAB 2018a. The key steps included [11, 12, 22]: (i) DICOM-to-NIFTI conversion, removal of the first 10 volumes for signal equilibrium, slice-timing correction, and realignment (excluding participants with translation > 2.5 mm or rotation > 2.5°; six DMD patients were excluded during this step); (ii) spatial normalization to Montreal Neurological Institute (MNI) space (resampled to 3 × 3 × 3 mm^3^); (iii) linear detrending and bandpass filtering (0.01–0.08 Hz) to reduce noise; (iv) removal of nuisance covariates (24 head motion parameters, global signal, white matter, and cerebrospinal fluid signals); and (v) scrubbing and despiking (via 3dDespike) to replace outliers with spline-interpolated values [12].

The intrinsic timescale was computed from preprocessed resting-state fMRI data. First, the autocorrelation function was calculated for the time series of each voxel [23]. The intrinsic timescale was defined as the positive area under the autocorrelation function curve—specifically, the sum of all positive autocorrelation coefficients from the starting point up to, but not including, the first lag where the autocorrelation becomes non-positive [10, 23]. This sum was multiplied by the repetition time to convert it into a temporal measure in seconds. This calculation was repeated for every voxel across the whole brain to generate a raw intrinsic timescale map. This process was implemented using MATLAB scripts from the RaichleLab GitHub repository (https://github.com/RaichleLab). For group-level statistical analyses, the map was spatially smoothed using a 6 mm full width at half maximum kernel to improve the signal-to-noise ratio and subsequently Z-transformed to standardize the data within groups.

#### Structural data preprocessing and GMV map

Voxel-based morphometry (VBM) was performed in SPM12 with the CAT12 toolbox (http://www.neuro.uni-jena.de/cat/) (MATLAB R2018a) to derive modulated GMV maps from T1-weighted structural MRI. The main steps were [11]: (i) segmentation of structural images into gray matter (GM), white matter (WM), and cerebrospinal fluid (CSF); (ii) spatial normalization to the MNI template (resampled to 2 × 2 × 2 mm^3^) and modulation; and (iii) smoothing of modulated GMV maps with a 6-mm full-width at half-maximum Gaussian kernel.

#### Statistical analysis

Demographic and clinical data, along with whole-brain volumes—including total intracranial volume (TIV), GM, WM, and CSF—were compared between the two groups using two-sample *t*-tests in IBM SPSS Statistics (v26.0), with the significance threshold set at *P* < 0.05.

We tested whether age-related trends in global volumetric measures (TIV, GM, WM, and CSF) differed between patients and controls using an ordinary least-squares linear model with a group × age interaction. For robustness, quadratic age terms and their interactions were initially added to the model and were removed due to non-significance; the final model retained only the linear age term and the group × age interaction. Analyses were conducted in GraphPad Prism v10.1.2 (Multiple Linear Regression). Evidence for different trends was based on the significance of the group × age interaction (two-tailed α = 0.05). Interaction-term *p*-values were multiplicity-adjusted using the Benjamini–Hochberg false discovery rate (FDR), with inference at *P* < 0.05.

Intrinsic timescale and GMV differences between the DMD and HC groups were tested with a two-sample *t*-test in the GLM framework based on SPM12 (http://www.fil.ion.ucl.ac.uk/spm/software/spm12/), controlling for age, years of education, duration of corticosteroid treatment, mean framewise displacement, and TIV. Multiple comparisons were controlled using Gaussian random-field theory (GRF) at a voxel-level threshold of *P* < 0.001, with a cluster-level significance threshold of *P* < 0.05, and the minimum cluster size of 22 voxels.

#### Correlation analysis

The cluster-wise mean intrinsic timescale and GMV values from regions showing significant group differences were extracted using the DPABI toolbox. Spearman rank correlations were then employed to explore the associations between these neural measures and the WISC-IV index scores (FSIQ, VCI, PRI, WMI, PSI). Multiple comparisons were corrected using the FDR method, with a significance threshold set at *P* < 0.05.

## Results

### Demographic and clinical characteristics

**Fig. 1:**
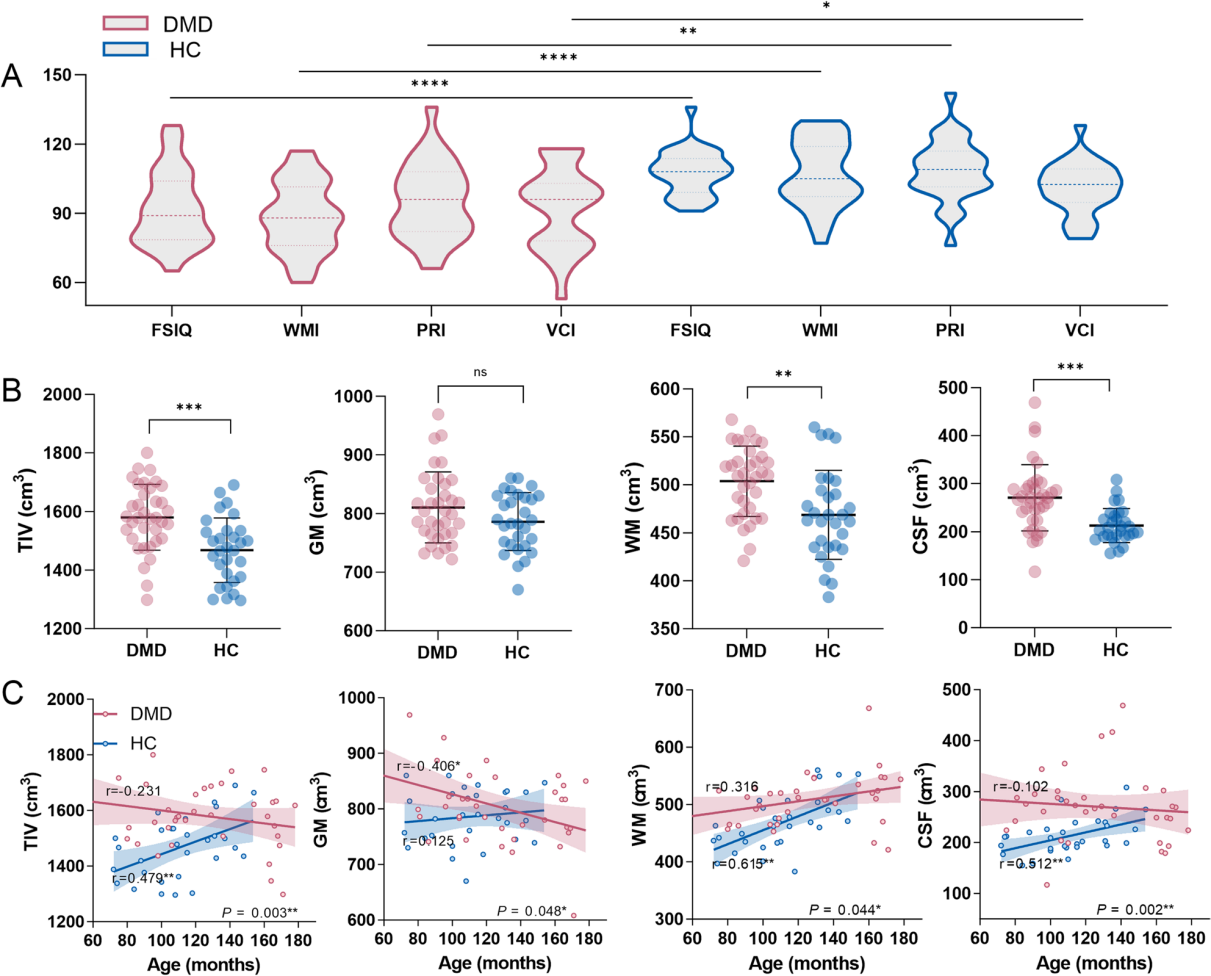
Differences in cognitive performance, global volumes, and brain structure–age-related trends between DMD and HC groups. **A** Compared with HCs, the DMD group exhibited significantly lower scores in FSIQ, VCI, PRI, and WMI. **B** Global structural volumes, including TIV, WM, and CSF, were significantly higher in the DMD group than in HCs. **C** There were significant group differences in age-related trend for all global volumetric measures. Group × age interaction *P* < 0.05 (FDR-corrected). Abbreviations: DMD, Duchenne muscular dystrophy; HC, healthy control; FSIQ, full-scale intelligence quotient; WMI, Working Memory Index; PRI, Perceptual Reasoning Index; VCI, Verbal Comprehension Index; TIV, total intracranial volume; GM, gray matter; WM, white matter; CSF, cerebrospinal fluid; FDR, False Discovery Rate. Significance symbol conventions are *: *P* < 0.05; **: *P* < 0.01; ***: *P* < 0.001; ns.: non-significant

This study included 36 children with DMD and 30 HCs; there were no significant group differences in age or years of education. Compared with HCs, the DMD group exhibited significantly lower scores in FSIQ, VCI, PRI, and WMI, as well as higher whole-brain structural volumes in TIV, WM, and CSF. There were significant group differences in age-related trend for all global volumetric measures (group × age interaction: TIV *P* = 0.003; GMV *P* = 0.048; WM *P* = 0.044; CSF *P* = 0.002, FDR-corrected). Specifically, TIV, GM, and CSF decreased with age in the DMD group but increased with age in controls, whereas WM increased in both groups, with a shallower slope in DMD than in controls. Further details are provided in Table 1; Fig. 1.

**Table 1:** Participant demographic and clinical characteristics

	DMD	HC	t	*P*-value
Sex, M/F	36/0	30/0	-	-
Age, years	9.50 ± 2.42	9.23 ± 2.08	0.475	0.637
Education, years	3.42 ± 2.22	2.90 ± 1.97	0.990	0.326
Duration of corticosteroid treatment, months	19.14 ± 15.20	-	-	-
Full-scale IQ	91.97 ± 16.30	107.70 ± 9.71	-4.484	< 0.001*
Verbal Comprehension Index	92.31 ± 17.71	100.77 ± 11.19	-2.185	0.034*
Perceptual Reasoning Index	95.28 ± 16.47	108.63 ± 13.28	-3.435	0.001*
Working Memory Index	88.62 ± 15.19	93.93 ± 12.83	-4.797	< 0.001*
Processing Speed Index	97.10 ± 17.39	107.13 ± 14.45	0.799	0.428
Total intracranial volume	1580.12 ± 112.04	1468.19 ± 110.20	4.071	< 0.001*
Gray matter	804.78 ± 68.39	786.23 ± 49.14	1.241	0.219
White matter	508.33 ± 45.22	468.83 ± 46.35	3.494	0.001*
Cerebrospinal fluid	270.53 ± 68.73	213.13 ± 35.55	4.360	< 0.001*

Abbreviations: *DMD* Duchenne muscular dystrophy, *HC* healthy control, *M* male, *F* female, *IQ* intelligence quotient

Values are presented as mean ± standard deviation. Significance symbol convention is *: *P* < 0.05

### Intrinsic timescale abnormalities in DMD versus HC

**Fig. 2:**
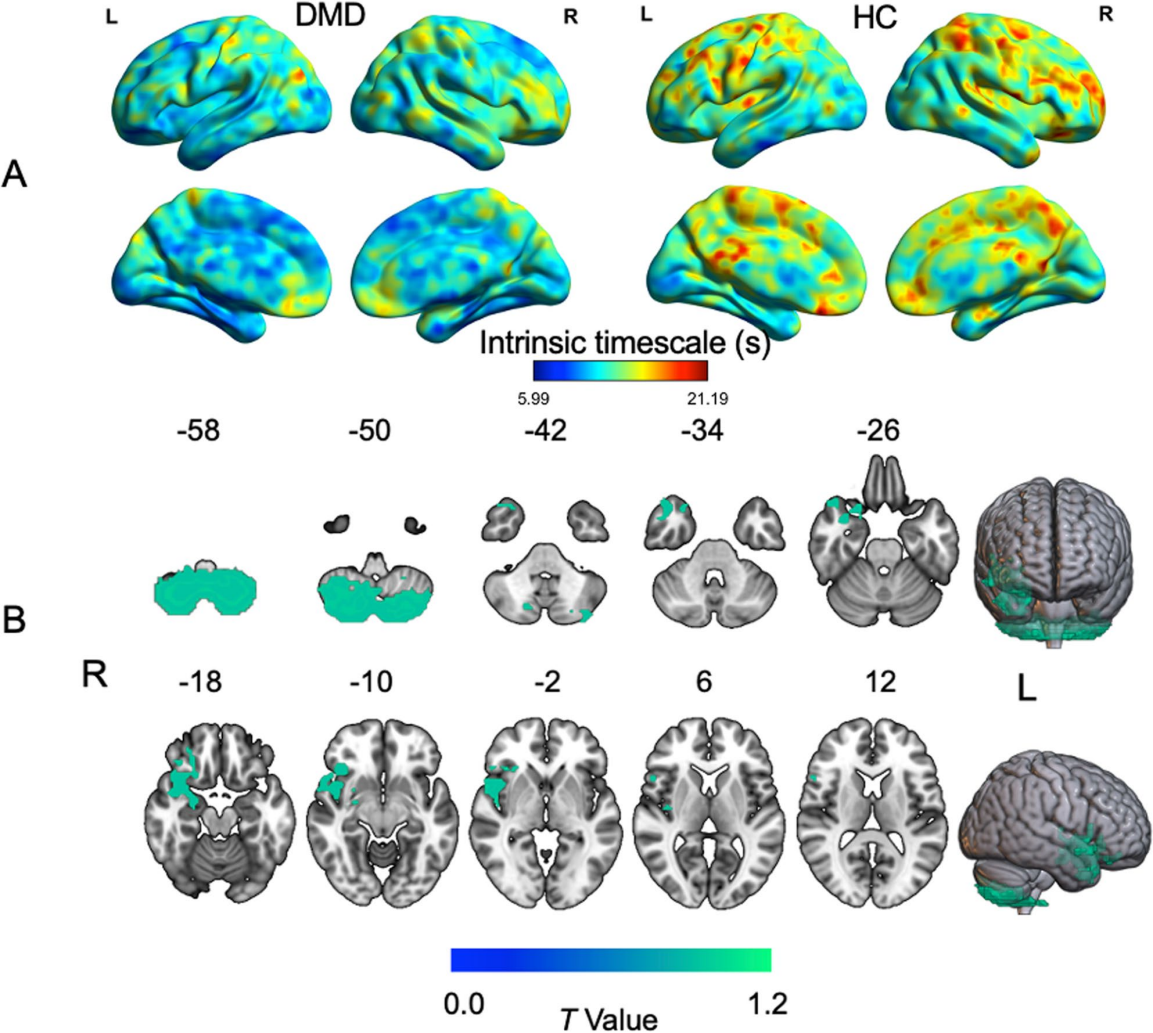
Intrinsic timescale differences between DMD and HC groups. **A** Spatial distribution maps of intrinsic timescales for children in the DMD and HC groups. At the whole-brain level, the intrinsic timescale was generally shorter in the DMD group than in the HC group. In both groups, the prefrontal lobe exhibited longer intrinsic timescales, whereas the occipital lobe showed shorter timescales. Warm colors indicate longer timescales, and cold colors indicate shorter timescales. **B** Voxel-wise differences in intrinsic timescales between the DMD and HC groups. The DMD group exhibited shorter intrinsic timescales in the bilateral CPL, right OFC, insula, and TPO extending to the hippocampus (GRF-corrected, voxel-wise P < 0.001, cluster-wise P < 0.05). Abbreviations: DMD, Duchenne muscular dystrophy; HC, healthy control; CPL, cerebellum posterior lobe; OFC, orbitofrontal cortex; TPO, temporal pole gyrus; colormap units, seconds.

Figure 2A presents the mean intrinsic timescale maps for the DMD and HC groups. Both groups exhibited comparable whole-brain patterns, such that timescales were longer in the prefrontal lobe and shorter in the occipital lobe. However, the DMD group demonstrated significantly shorter intrinsic timescales in the bilateral cerebellum posterior lobe (CPL), right orbitofrontal cortex (OFC), insula, and temporal pole gyrus (TPO), extending to the hippocampus (Table 2, Fig. 2B).

**Table 2:** Voxel-wise comparisons of intrinsic timescale and GMV between DMD and HC groups

Brain region		Hemisphere	Peak MNI coordinate	Cluster size (voxels)	Peak T value
			(X, Y, Z)		
Intrinsic timescale					
DMD < HC					
CPL		Bilateral	18, -54, -63	1826	-7.25
TPO+Hippocampus		Right	44, 3, -15	245	-4.1
Insula		Right	38, 24, -7	115	-3.92
OFC		Right	34, 24, -7	99	-3.9
DMD > HC					
None					
GMV					
DMD < HC					
CPL		Right	21, -60, -65	1504	-6.34
CPL		Left	-39, -54, -62	2018	-6.29
OFC + TPO		Right	35, 21, -21	994	-6.04
Insula		Right	57, 11, -2	868	-5.23
Insula		Left	-42, 12, -2	617	-5.55
MTG		Left	-65, -30, -3	1101	-6.22
Thalamus		Bilateral	9, -15, 20	1669	-5.88
Precuneus		Right	12, -75, 41	785	-4.68
Precuneus		Left	-20, -72, 54	1331	-5.65
Occipital cortex		Left	-27, -86, 31	239	-4.32
DMD > HC					
DPFC		Right	14, 2, 69	2275	5.96
DPFC		Left	-17, 5, 69	969	4.88

*Abbreviations*: *GMV* gray matter volume, *DMD* Duchenne muscular dystrophy, *HC* healthy control, *CPL* cerebellum posterior lobe, *TPO* temporal pole gyrus, *OFC* orbitofrontal cortex, *MTG* middle temporal gyrus, *DPFC* dorsal prefrontal cortex

### GMV abnormalities in DMD versus HC

**Fig. 3:**
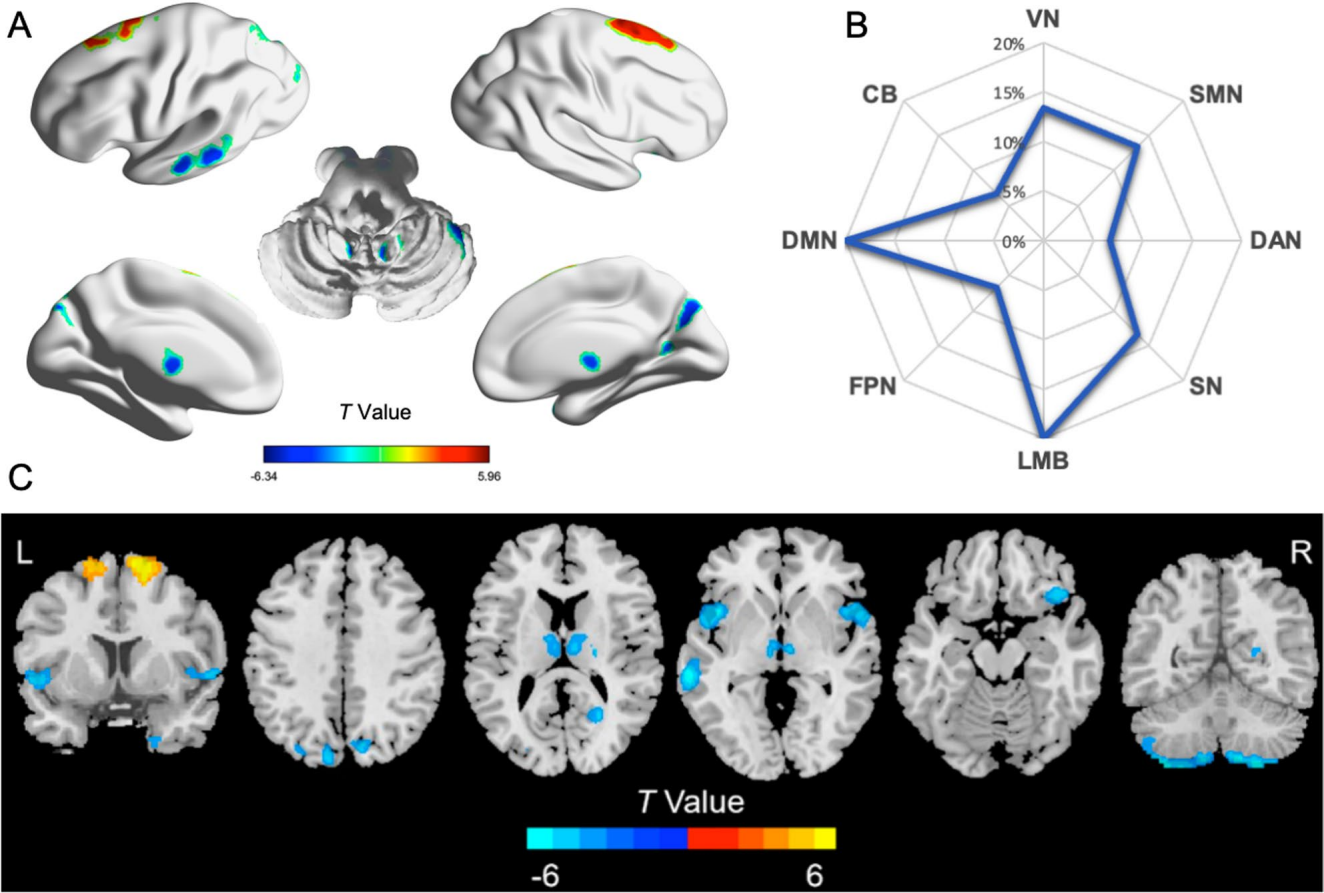
GMV differences between DMD and HC groups. **A** Voxel-wise GMV differences between the DMD and HC groups. Compared with HCs, the DMD group exhibited lower GMV in the bilateral CPL, insula, thalamus, precuneus, right OFC/TPO, and left MTG; it showed higher GMV in the DPFC (GRF-corrected, voxel-wise *P* < 0.001, cluster-wise *P* < 0.05). Warm and cold colors denote higher and lower GMV in DMD, respectively. **B** Brain regions with significant GMV differences were localized to large-scale neural networks, predominantly the DMN and LMB. **C** Voxel-wise GMV differences between DMD and HC groups displayed in volumetric space. Abbreviations: GMV, gray matter volume; DMD, Duchenne muscular dystrophy; HC, healthy control; CPL, cerebellum posterior lobe; OFC, orbitofrontal cortex; TPO, temporal pole gyrus; MTG, middle temporal gyrus; DPFC, dorsal prefrontal cortex; DMN, default mode network; CB, cerebellum; VN, visual network; SMN, sensorimotor network; DAN, dorsal attention network; SN, salience network; LMB, limbic system; FPN, frontoparietal network; WISC-IV, Wechsler Intelligence Scale for Children–Fourth Edition; L, left; R, right

Compared with HCs, the DMD group exhibited lower GMV in the bilateral CPL, right OFC, right TPO, bilateral insula, left MTG, bilateral thalamus, and precuneus; it displayed higher GMV in the dorsal prefrontal cortex (DPFC) (GRF-corrected, voxel-wise *P* < 0.001, cluster-wise *P* < 0.05; Table 2; Fig. 3).

### Correlations of GMV and intrinsic timescale with WISC-IV scores in the DMD group

**Fig. 4:**
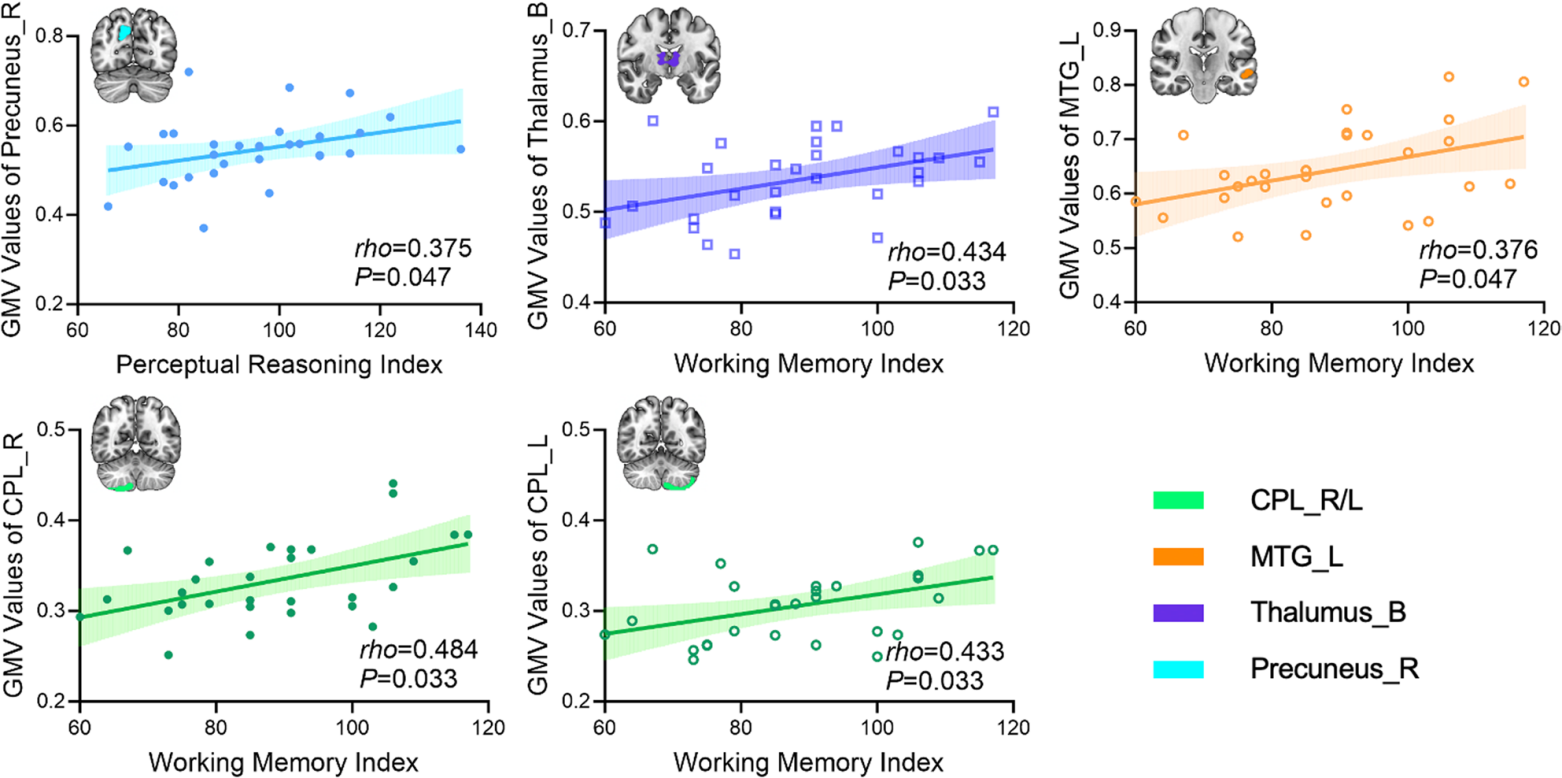
Correlations between GMV and WISC-IV scores in the DMD group. Solid and hollow circles indicate right and left hemispheric regions, respectively; colors distinguish brain regions. Significance threshold set at *P* < 0.05 (FDR-corrected). Abbreviations: GMV, gray matter volume; DMD, Duchenne muscular dystrophy; HC, healthy control; CPL, cerebellum posterior lobe; MTG, middle temporal gyrus; WISC-IV, Wechsler Intelligence Scale for Children–Fourth Edition; L, left; R, right; FDR, False Discovery Rate

GMV in the bilateral CPL, thalamus, and left MTG was positively associated with WMI scores, and GMV in the right precuneus was positively associated with PRI scores (FDR-corrected, *P* < 0.05) (Fig. 4). No significant correlations between intrinsic timescale and WISC-IV scores were observed in the DMD group.

## Discussion

This study integrated advanced methodologies of INT and VBM to evaluate the temporal properties of brain activity and morphological alterations in children with DMD, and examined how these abnormalities relate to cognition. We observed broad neurocognitive deficits and group differences in age-related trends of global volumetric measures relative to controls. Compared with HCs, the DMD group showed co-located INT shortening and GMV reductions within limbic–sensorimotor networks; additionally, there were widespread GMV alterations in the visual network, DMN, and dorsal attention network, alongside domain-specific structure–cognition associations.

Prior work indicates multidomain cognitive impairment in children with DMD—including reduced FSIQ and deficits in memory, language processing, and perceptual reasoning [6, 24]—which accords with our observation of lower FSIQ, VCI, PRI, and WMI scores in the DMD group relative to HCs. In addition, the DMD group exhibited abnormalities in global volumetrics: TIV, WM, and CSF were higher than in HCs, whereas total GM did not differ significantly between groups. This pattern suggests that elevated TIV is primarily driven by WM expansion and CSF enlargement. Although increased TIV might superficially imply preserved brain growth, CSF enlargement may reflect compensatory ventriculomegaly in the context of cortical thinning, a configuration reminiscent of neurodegenerative processes described in Huntington’s disease [25]. WM augment in DMD may reflect abnormal myelination or reactive gliosis [26]. The absence of a group difference in total GM likely reflects regional heterogeneity, with GMV increases in some areas offsetting decreases in others—as supported by our VBM findings showing widespread network-level GMV reductions coexisting with relative enlargement in the DPFC. Moreover, age-related trends of global volumes diverged between groups: in DMD, TIV/GMV/CSF decreased with age, whereas these measures increased with age in HCs; WMV increased in both groups, but the slope was shallower in DMD. Taken together, these patterns indicate disruption of neurodevelopmental programming.

We observed co-localized INT shortening and GMV reductions in the CPL, OFC, insula, and TPO, consistent with prior evidence that regions with lower GMV tend to exhibit shorter INT [23]. This pattern accords with the hypothesis that reduced capacity to sustain and integrate information co-occurs with compromised structural integrity in DMD. At the whole-brain level, both groups preserved the canonical INT gradient [27]—longer timescales in higher-order cortices (e.g., prefrontal) and shorter timescales in the occipital visual cortex [28, 29]—supporting a hierarchical organization in which integration windows expand from primary to association areas [30].

Beyond its classical roles in motor coordination and balance, the cerebellum contributes to higher-order cognitive control and visuomotor processing [8]. In DMD, dystrophin loss in Purkinje cells disrupts GABA_A_ receptor clustering [16], reduces inhibitory synaptic function, and alters postsynaptic currents [17]; such GABAergic impairments have been linked to cognitive and behavioral abnormalities in mdx mice and in patients with DMD [31]. The OFC, insula, TPO, and hippocampus are integral to limbic–behavioral regulation, supporting emotion regulation, behavioral flexibility, memory, and learning [32]. The OFC integrates reward and punishment signals to guide decision-making [33], whereas the insula mediates interoceptive awareness and emotional salience [34]. Structural–functional compromise in these regions may underlie anxiety and social withdrawal reported in DMD [35]. The TPO, a hub for social cognition and semantic memory [36], showed concurrent INT and GMV reductions, aligning with behavioral reports of theory-of-mind and emotion-recognition impairments in DMD [37]. Given its broad connectivity with the DMN and limbic structures, TPO dysfunction may disrupt social information integration, thereby amplifying psychosocial challenges in DMD [38].

In addition to the co-located regions, we observed widespread GMV reductions in the occipital cortex, precuneus, MTG, and thalamus, whereas the DPFC showed increased GMV. This pattern indicates that DMD can exhibit a dual profile of regional GMV decreases and increases. The precuneus, a core hub of the default mode network (DMN) involved in self-referential processing and episodic memory [39] and in integrating sensory inputs with mental representations critical for perceptual reasoning [40], showed reduced GMV that was positively associated with PRI. This underscores its role in visuospatial buffering and mental imagery and suggests that preserving precuneus structural integrity may help mitigate related visuospatial and reasoning deficits.

WMI scores were correlated with GMV in the left MTG, bilateral CPL, and thalamus. The thalamus—a relay linking the striatum, cortex, and cerebellum—modulates prefrontal activity, integrates sensorimotor signals, and enhances information transfer essential for task maintenance [41]. Its reduction can disrupt corticostriatal circuits, exacerbating executive function deficits in DMD [2]. Additionally, the MTG, which is vital for language and semantic memory [42], exhibited lower GMV. Degeneration in this region may underlie the language delays and verbal memory deficits encountered in DMD patients [24]. Its role in phonological processing and the contribution of the cerebello-thalamo-cortical circuit to working memory imply that the integrity of these regions supports verbal working memory [43]. These correlations suggest that interventions aimed at preserving GMV in regions such as the CPL, MTG, and precuneus could mitigate cognitive decline. For instance, cognitive training programs designed to enhance working memory and visuospatial skills may stimulate neuroplasticity in these regions. GMV reduction in the occipital cortex corresponds with reports of visual-perceptual deficits in DMD [37]. Given the occipital lobe’s role in visual processing and its connectivity with parietal attentional networks, it may be particularly vulnerable to dystrophin deficiency-induced disruptions, potentially leading to visuospatial impairment [44].

In contrast to widespread GMV reduction in DMD, the DPFC, a key region of the frontoparietal and dorsal attention networks involved in executive control, working memory, decision-making, attention regulation, cognitive flexibility, and social cognition [45], exhibited increased GMV. The finding is compatible with compensatory neuroplasticity, but remains speculative. In Alzheimer’s disease, similar GMV increases have been associated with gliosis or compensatory neurogenesis [11]. In DMD, chronic neuroinflammation driven by dystrophin-deficient microglia may induce reactive astrocytosis, which transiently increases GMV but may compromise functional efficiency [2]. However, the adaptive or maladaptive nature of this hypertrophy requires exploration through longitudinal studies. We need to examine, longitudinally, whether the growth rate of the DPFC predicts maintenance or improvement of executive function, and, cross-sectionally, whether greater DPFC volume is associated with better executive/working-memory performance.

Although this study provided novel insights, several limitations warrant consideration. First, the cross-sectional design precludes causal inferences regarding relationships among GMV, intrinsic timescale, and cognition; group × age effects should be interpreted as between-group differences in age-related trends, not within-subject longitudinal change. Longitudinal studies tracking disease progression are needed. Second, although the sample size is acceptable for a rare disease, statistical power remains limited; replication in larger cohorts is warranted. Third, data limitations restricted neurocognitive analyses to the cohort-wide DMD population, precluding dystrophin subtype-specific genotype-phenotype correlations. Finally, the single-center pediatric sample may limit generalizability, underscoring the need for multi-center replication across developmental stages.

## Conclusion

This study revealed multilevel neural abnormalities in DMD: within limbic–sensorimotor networks we observed co-localized deficits in the temporal properties of brain activity and in morphology, alongside widespread GMV alterations associated with cognitive impairment and divergent age-related volumetric trends. These findings underscore the pivotal role of dystrophin deficiency in the neurodevelopmental program of DMD. By INT with VBM, this work established a multimodal framework for dissecting neurodevelopmental pathology and prioritized testable, potentially actionable targets for intervention.

## Data Availability

The datasets generated and/or analyzed during the current study are not publicly available due to confidentiality but are available from the corresponding author on reasonable request.

## Abbreviations

DMD: Duchenne muscular dystrophy
INT: Intrinsic neural timescale
MRI: Magnetic resonance imaging
rs-fMRI: Resting-state functional magnetic resonance imaging
VBM: Voxel-based morphometry
GMV: Gray matter volume
FSIQ: Full-scale intelligence quotient
DMN: Default mode network
ECN: Executive control network
HCs: Healthy controls
WISC-IV: Wechsler Intelligence Scale for Children-Fourth Edition
VCI: Verbal comprehension index
PRI: Perceptual reasoning index
WMI: Working memory index
PSI: Processing speed index
3D-MPRAGE: Three-dimensional magnetization-prepared rapid gradient echo
DPARSF: Data Processing Assistant for Resting-State fMRI
SPM 12: Statistical Parametric Mapping 12
MNI: Montreal Neurological Institute
CAT 12: Computational Anatomy Toolbox 12
GM: Gray matter
WM: White matter
CSF: Cerebrospinal fluid
TIV: Total intracranial volume
GRF: Gaussian random field
CPL: Cerebellum posterior lobe
OFC: Orbitofrontal cortex
TPO: Temporal pole gyrus
DPFC: Dorsal prefrontal cortex
